# CPH-I and HE4 Are More Favorable Than CA125 in Differentiating Borderline Ovarian Tumors from Epithelial Ovarian Cancer at Early Stages

**DOI:** 10.1155/2019/6241743

**Published:** 2019-10-13

**Authors:** Zhiheng Wang, Xiang Tao, Chunmei Ying

**Affiliations:** Obstetrics and Gynecology Hospital of Fudan University, China

## Abstract

**Aim:**

To evaluate the diagnosis value of serum human epididymis protein 4 (HE4), cancer antigen 125 (CA125), the Risk of Ovarian Malignancy Algorithm (ROMA), and Copenhagen Index (CPH-I) at early stages for differentiating borderline ovarian tumors from epithelial ovarian cancer.

**Methods:**

We recruited 144 borderline ovarian tumors in FIGO stages I and II (BOT I+II), 108 epithelial ovarian cancers in FIGO stages I and II (EOC I+II), and 238 benign ovarian tumor patients with surgical treatment in the retrospective study. The concentration of HE4 and CA125 and the values of CPH-I and ROMA were assessed separately.

**Results:**

The HE4 level and ROMA and CPH-I values of EOC I+II were all higher than that of BOT I+II and benign groups whether in all, pre-, or postmenopausal groups (*P* < 0.01). When distinguishing BOT I+II from EOC I+II, the AUC-ROC of CPH-I and HE4 were bigger than CA125 (*P* < 0.001), while the CPH-I has the highest sensitivities in all and postmenopausal groups (78.7%, 85.1%), and HE4 has the highest specificity and PPV (90.91%, 88.64%) in postmenopausal groups. Under pathological stratification, HE4, ROMA, and CPH-I of the serous EOC I+II were higher than that of BOT I+II (*P* < 0.001) and the AUC of the three indices were significantly bigger than CA125 (*P* < 0.001). However, the concentration of HE4 and CA125 and the values of CPH-I and ROMA have no significant difference between the two endometrioid subgroups. The index with the highest sensitivity and NPV among the four indices of different pathological subtype groups was CPH-I, and the index with the highest specificities and PPV was HE4.

**Conclusion:**

CPH-I was more valuable than CA125 for differentiating BOT I+II from EOC I+II regardless of menopausal status, while HE4 might be better than CA125 for postmenopausal subgroups. HE4 and CPH-I were more favorable than CA125 for differentiating BOT I+II from EOC I+II in the case of unknown pathology or in serous type.

## 1. Introduction

The importance of preoperative differentiation between the borderline ovarian tumors (BOT) and epithelial ovarian cancer (EOC) was gradually recognized [1, 2]. BOT, defined with low malignant potential, differ from EOC by many characters such as etiology, expression of tumor markers, and prognosis [3]. Early identification of BOT and EOC is very important regarding the different treatments of both, such as the extent and method of surgery, the need of preserved fertility function for women, and the need of postoperative chemotherapy and infertility treatments [4–6].

However, the preoperational differentiation between BOT and EOC has always been a clinical difficulty as abdominal distension and abdominal pain may appear in both of them and imaging cannot accurately and effectively identify both, especially in their early stages [7]. Studies have shown that the accurate diagnosis rate of ultrasound for BOT of all stages was only 69% [8] and the specificity of MRI for diagnosis of BOT was only 45.4% [9], although imaging could identify ovarian cancer from benign tumors well [10]. The accuracy of common clinically used intraoperative frozen section methods on BOT has been confirmed to be only about 60% [11]. The delayed postoperative definitive pathology may increase the possibility of reoperation and delay in adjuvant treatment and may even lead to the possibility of tumor spread [11, 12].

Therefore, it is particularly necessary to find sensitive and specific indicators to differentiate BOT from EOC before clinical operation, which may be helpful for pathologists. CA125, HE4, and ROMA have been widely used in the differentiation of EOC and benign tumors [13–15], although they were affected by the menopausal status [16]. CPH-I, a new tumor index calculated by HE4, CA125, and age rather than menopausal status with different definitions [15, 17, 18], was thought to be easier to obtain than ROMA clinically [15]. Researches have shown that CPH-I and ROMA have similar discriminatory performance in benign lesions and malignant ovarian tumors [15, 19]. Studies have also shown that CA125 and HE4 have different manifestations in different pathological types and subtypes of ovarian tumors [20, 21]. However, few studies focus on the identification of BOT and EOC by using CA125, HE4, ROMA, and CPH-I and the differences of the four indices in similar pathological subtypes of BOT and EOC, especially in the early stages and different menopausal statuses.

This retrospective study assessed the diagnosis value of serum HE4, CA125, ROMA, and CPH-I in patients with an ovarian tumor in early stages based on menopausal status and similar pathological subtypes, aimed at finding more accurate and effective markers for BOT and EOC.

## 2. Materials and Methods

### 2.1. Patients

We analyzed all patients with an ovarian tumor who underwent surgery and were diagnosed by pathology in the Obstetrics and Gynecology Hospital of Fudan University from May 2015 to April 2018. The serum levels of HE4 and CA125 of all patients were measured within four weeks before surgery. The patients were staged (I–IV) according to the International Federation of Gynecology and Obstetrics (FIGO) 2014 standards. Exclusion criteria were another benign or malignant tumors, ovarian tumors treated by preoperative chemotherapy, gynecological diseases (e.g., endometriosis and pelvic infection), liver disease (hepatic dysfunction), renal disease (abnormal serum creatinine), lung disease, heart failure, autoimmune diseases (e.g., systemic lupus erythematosus), or pancreatitis (amylase enzyme abnormalities).

A total of 490 individuals were finally enrolled, including 108 EOC patients in FIGO stages I and II (EOC I+II, aged 16–74 years), 144 BOT patients in FIGO stages I and II (BOT I+II, aged 13-71 years), and 238 benign ovarian tumor patients who were randomly selected (benign, aged 18-68 years).

The standard for patients diagnosed as postmenopausal was no menstruation within 12 months; otherwise, they were considered premenopausal. All pathological sections were diagnosed by two or more professional pathologists and further classified the EOC and BOT into serous, mucinous, endometrioid, and clear cell types. The characteristics of the 490 participants and histopathological diagnoses were shown in Table 1.

### 2.2. Detection Methods

Serum levels of HE4 and CA125 were measured using a Roche COBAS e 601 electrochemiluminescence analyzer (Roche Diagnostics Ltd., Switzerland).

ROMA was calculated using the following equations [22, 23]:

(1) $$\begin{array}{c} \text{Premenopausal}:\text{Predictive Index }\left(\text{PI}\right)=-12.0+2.38*\text{LN}\left(\text{HE}4\right)+0.0626*\text{LN}\left(\text{CA}125\right),\\ \text{Postmenopausal}:\text{Predictive Index }\left(\text{PI}\right)=-8.09+1.04*\text{LN}\left(\text{HE}4\right)+0.732*\text{LN}\left(\text{CA}125\right),\\ \text{Predicted Probability }\left(\text{PP}\right)=\frac{\exp\left(\text{PI}\right)}{1/\exp\left(\text{PI}\right)}. \end{array}$$

CPH-I was calculated using the following equations [17]:

(2) $$\begin{array}{c} \text{CPH}‐\text{I}=-14.0647+1.0649*\log_{2}\left(\text{HE}4\right)+0.6050*\log_{2}\left(\text{CA}125\right)+0.2672*\frac{\text{age}}{10},\\ \text{PP}=\frac{e^{\text{CPH}‐\text{I}}}{1+e^{\text{CPH}‐\text{I}}}. \end{array}$$

Comparative analysis of HE4, CA125, ROMA, and CPH-I was conducted within groups and subgroups. Sensitivity, specificity, positive predictive value (PPV), and negative predictive value (NPV) of HE4, CA125, ROMA, and CPH-I in the identification of BOT I+II and EOC I+II were calculated by the MedCalc statistical program in different menopausal statuses and histopathological subtypes. The diagnostic value of each marker was assessed by the area under the receiver operating characteristic curve (AUC-ROC).

### 2.3. Statistical Analysis

The data was expressed as the median (interquartile range). The differences of HE4 and CA125 levels and ROMA and CPH-I values among groups were compared using the Mann–Whitney *U* test or Kruskal-Wallis one-way ANOVA test by SPSS (Version 20.0; IBM, NY, US), and all reported *P* values were two-tailed. The ROC curve analysis has been determined using the MedCalc statistical program. *P* < 0.05 was considered to be of statistical significance.

## 3. Results

### 3.1. General Information and Comparison of HE4, CA125, ROMA, and CPH-I among Groups

In the baseline character of the 490 participants, the age of EOC I+II was older than benign and BOT I+II groups (*P* < 0.001) with no statistically significant difference between benign and BOT I+II groups (*P* = 1.00) (Table 1). The HE4 and CA125 levels and ROMA and CPH-I values all showed the most pronounced increase in EOC I+II, followed by BOT I+II, and the lowest in the benign group (*P* < 0.009) (Table 2) and the same trend with the four indices of premenopausal patients (Pre-M) (*P* < 0.025). However, the four indices in postmenopausal patients (Post-M) were somehow different. The HE4 and CA125 levels and ROMA and CPH-I values in EOC I+II Post-M were significantly higher than that of BOT I+II and benign corresponding subgroups (*P* < 0.002), and levels of CA125 in EOC I+II Post-M were only higher than that of the benign subgroup (*P* < 0.001). CA125 levels in EOC I+II Post-M had no statistical difference compared with the BOT I+II Post-M subgroup (*P* = 0.054).

### 3.2. ROC Curve Analysis of Diagnostic Values of HE4, CA125, ROMA, and CPH-I for Differentiating EOC I+II and BOT I+II in Patients of All or under Different Menopausal Status Groups

**Figure 1:**
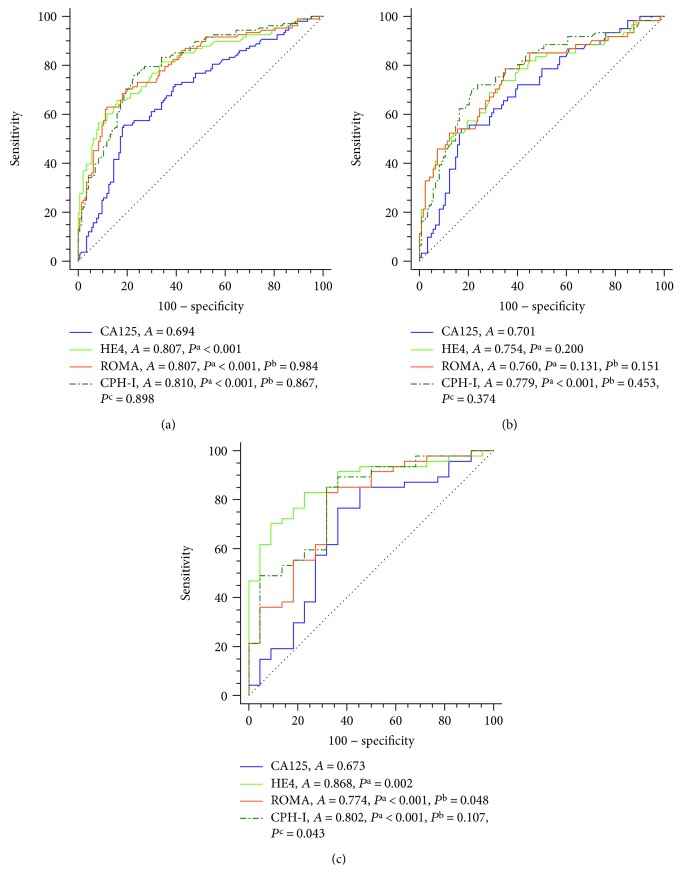
ROC curve analysis of the diagnostic values of CA125 and HE4 levels and ROMA and CPH-I indices for BOT I+II and EOC I+II of all patients (a), premenopausal patients (b), and postmenopausal patients (c). Abbreviations: Pre-M = premenopausal; Post-M = postmenopausal; *A* = AUC-ROC; *P*^a^, *P*^b^, and *P*^c^ = *P* value of AUC-ROC compared with CA125, HE4, and ROMA, respectively.

The ROC curve analyses were conducted to compare the diagnostic values of CA125, HE4, ROMA, and CPH-I for differentiating EOC I+II from BOT I+II in patients of all or under different menopausal statuses (Figure 1). As shown in Figure 1, the AUC of CPH-I (AUC = 0.81), HE4 (AUC = 0.81), and ROMA (AUC = 0.81) were significantly bigger than CA125 (AUC = 0.69) when differentiating BOT I+II from EOC I+II (*P* < 0.001, *P* < 0.001, and *P* < 0.001, respectively). In Pre-M patients, the AUC of CPH-I (AUC = 0.78) was statistically bigger than CA125 (AUC = 0.70) (*P* < 0.001), while there was no significant difference among the AUC of HE4, CA125, and ROMA. In Post-M patients, the AUC of HE4, CA125, ROMA, and CPH-I were 0.87, 0.67, 0.77, and 0.80, respectively. The AUC of HE4, ROMA, and CPH-I were significantly bigger than CA125 (*P* = 0.002, *P* < 0.001, and *P* < 0.001, respectively). Moreover, the AUC of both HE4 and CPH-I were bigger than ROMA (*P* = 0.048 and *P* = 0.043, respectively) with no significant difference between the AUC of HE4 and CPH-I.

Those data suggested that the overall diagnostic values of HE4, ROMA, and CPH-I were better than CA125 in differentiating EOC I+II from BOT I+II, and HE4 and CPH-I had the best predictive value in the Post-M group.

### 3.3. Sensitivity, Specificity, PPV, and NPV of HE4, CA125, ROMA, and CPH-I in Patients of All or under Different Menopausal Status Groups

The BOT I+II and EOC I+II groups and pre- and postmenopausal subgroups were considered in this investigation. The sensitivity, specificity, PPV, and NPV of HE4, CA125, ROMA, and CPH-I of different groups were shown in Table 3. In all patients, the HE4, CA125, ROMA, and CPH-I sensitivities in the diagnostic of EOC I+II from BOT I+II were 65.74%, 55.56%, 62.96%, and 78.70% and the specificities were 84.03%, 81.25%, 88.19%, and 74.31%, respectively. The PPV of HE4 (75.53%) was more favorable than that of other indices, and CPH-I has the highest NPV (82.30%). In the Pre-M groups, both ROMA sensitivity (78.69%) and NPV (85.71%) were the highest among all indices, while CA125 (82.79%) had the highest specificity, and HE4 (64.00%) had the highest PPV. However, in the Post-M groups, CPH-I showed the highest sensitivity (85.11%) and NPV (68.20%), while HE4 had the highest specificity (90.91%) and PPV (88.64%).

### 3.4. Comparison of HE4, CA125, ROMA, and CPH-I in Patients with Different Pathological Subtypes between BOT I+II and EOC I+II

The HE4 and CA125 levels and ROMA and CPH-I values of four pathological subtype groups were shown in Table 4. The statistical analysis of the four indices of the clear cell tumors was not performed to prevent bias as there were only four clear cell BOT I+II cases. Levels of HE4 (*P* < 0.001 and *P* = 0.004, respectively) and values of ROMA (*P* < 0.001 and *P* = 0.010, respectively) and CPH-I (*P* < 0.001 and *P* = 0.002, respectively) in serous and mucinous EOC I+II subgroups were all higher than that of BOT I+II corresponding subgroups. Levels of CA125 were significantly higher only in serous EOC I+II than serous BOT I+II (*P* < 0.001). There was no significant difference between endometrioid subgroups with HE4, CA125, ROMA, and CPH-I (*P* = 0.071, 0.458, 0.112, and 0.183, respectively).

### 3.5. ROC Curve Analyses of Diagnostic Values of HE4, CA125, ROMA, and CPH-I for Differentiating EOC I+II from BOT I+II in Patients with Different Pathological Subtypes

**Figure 2:**
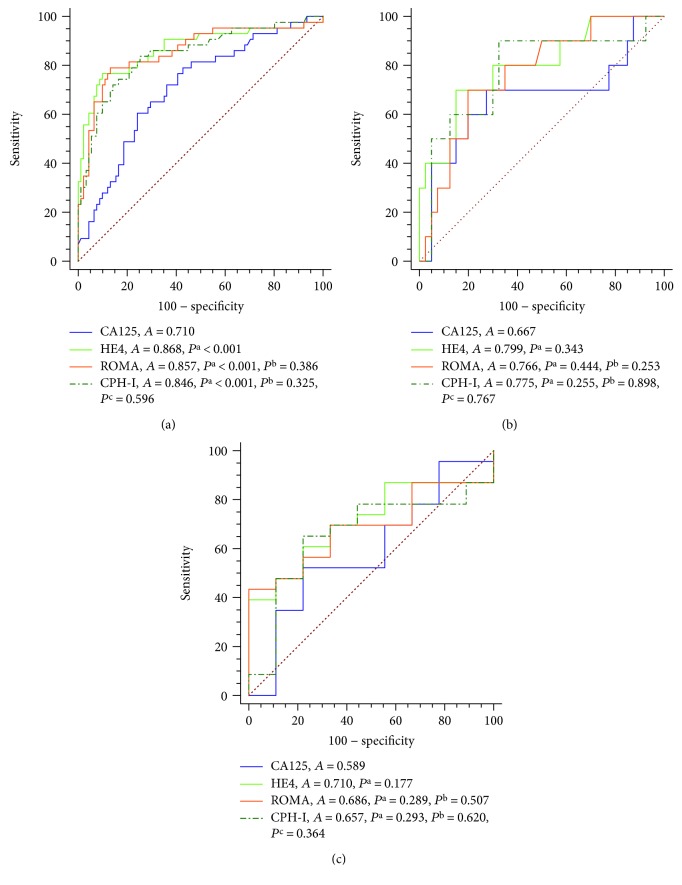
ROC curve analyses of the diagnostic values of CA125 and HE4 levels and ROMA and CPH-I indices for BOT I+II and EOC I+II in serous (a), mucinous (b), and endometrioid (c) tumors. Abbreviations: *A* = AUC-ROC; *P*^a^, *P*^b^, and *P*^c^ = *P* value of AUC-ROC compared with CA125, HE4, and ROMA, respectively.

The ROC curve analyses were also performed in patients with different pathological subtypes in BOT I+II vs. EOC I+II groups (Figure 2). In patients with serous tumors, the AUC of HE4, CA125, ROMA, and CPH-I were 0.87, 0.71, 0.86, and 0.85, respectively. The AUC of HE4, ROMA, and CPH-I were remarkably bigger than that of CA125 (*P* < 0.001, *P* < 0.001, and *P* < 0.001, respectively). In patients with the mucinous and endometrioid tumors, there was no significant difference in the AUC among the four indices.

These data demonstrated that the diagnostic values of HE4, ROMA, and CPH-I were better than that of CA125 in patients with a serous tumor type in differentiating EOC I+II from BOT I+II.

### 3.6. Sensitivity, Specificity, PPV, and NPV of HE4, CA125, ROMA, and CPH-I in Patients with Different Pathological Subtypes

The serous, mucinous, and endometrioid tumor groups were considered in this investigation; the sensitivity, specificity, PPV, and NPV of HE4, CA125, ROMA, and CPH-I of different pathological subtype groups were shown in Table 5. The highest sensitivity of the four indices reached 93.75% (CPH-I) in the mucinous tumor group, while the lowest was 39.13% (HE4) in the endometrioid tumor group. The highest specificity of the four indices reached 100% (HE4) in the endometrioid tumor group, while the lowest was 67.5% (CPH-I) in the endometrioid tumor group. The CPH-I sensitivity and NPV in serous (83.72% and 90.7%), mucinous (90% and 96.4%), and endometrioid (65.22% and 46.7%) were the highest among the four indices. The HE4 specificities and PPV in serous (90.11% and 78.57%), mucinous (85% and 53.85%), and endometrioid (100% and 100%) were the highest among the four indices.

These data suggested that the combination of HE4 and CPH-I might have a better effect on distinguishing the corresponding pathological types of EOC I+II from BOT I+II.

## 4. Discussion

The results of the present retrospective study demonstrate that CA125, HE4, ROMA, and CPH-I were useful for differentiating BOT I+II from EOC I+II, with the overall diagnostic value of HE4, ROMA, and CPH-I better than CA125, while in the Post-M group, HE4 and CPH-I had the best predictive value. When used to distinguish serous subtype of BOT I+II from that of EOC I+II, the diagnostic values of HE4, ROMA, and CPH-I were better than that of CA125, with no significant difference of the four indices between the two endometrioid subgroups. As far as we know, it was the first study to compare the diagnosis value of CPH-I with the other indices in differentiating BOT I+II from EOC I+II under pre- and postmenopausal statuses and between their similar pathological subtypes.

It is difficult to compare the performance of the CA125, HE4, ROMA, and CPH-I across different studies since only a few studies compared the four indices simultaneously in differentiating BOT from EOC, and these studies were mostly with a small number of cases and did not focus on the early stage of BOT and different pathological subtypes.

Studies showed that levels of HE4 and CA125 and the ROMA index were all higher in EOC I-IV than that of BOT I-IV [16, 20, 24, 25], with other two studies reporting the same trend for CPH-I [15, 19]. Moreover, Kotowicz et al. showed that levels of HE4 and CA125 and the ROMA index were all higher in EOC I+II than BOT I-IV [26]. In our study, we also found that the four indices were all higher in EOC I+II than that of BOT I+II. In addition, we found that the four indices were all higher in premenopausal or postmenopausal EOC I+II subgroups than that of BOT I+II but with no statistical difference of CA125 between postmenopausal subgroups. However, Zhang et al. reported that HE4, CA125, and ROMA were higher in EOC I-IV (*n* = 181) than BOT I-IV (*n* = 17) whether in premenopausal or postmenopausal subgroups [20], and Chudecka-Glaz et al. have a similar finding but with no statistical difference of HE4 when comparing BOT I-IV (*n* = 11) with EOC I-IV (*n* = 38) under premenopausal [24]. These inconsistent results might be mainly due to the different participants' numbers and FIGO stages, and we focused on the early stages of BOT and EOC in more participants.

With regard to the diagnostic value of the four indices, we found that the AUC-ROC of HE4, ROMA, and CPH-I were bigger than that of CA125 (0.807, 0.807, and 0.810 vs. 0.694, *P* < 0.001). However, our AUC of ROMA and CPH-I were much higher than that reported in Minar et al.'s study (0.65 and 0.67, respectively) when distinguishing 42 BOT I+II patients from 33 EOC I+II patients [15]. The difference maybe mainly due to different races which may cause different proportions of pathological subtypes in ovarian tumors [27–29], further resulting in different ROMA and CPH-I [20, 21]. We also found that the AUC of HE4 and CPH-I were mostly significantly higher than that of CA125 in patients of postmenopausal subgroup, with only CPH-I bigger than that of CA125 in premenopausal. Moreover, CPH-I has higher sensitivity and NPV in all subgroups. Therefore, CPH-I was outstandingly valuable than CA125 in differentiating BOT I+II and EOC I+II of all patients regardless of menopausal status, while HE4 might be better than CA125 for patients of all or postmenopausal subgroups, and ROMA was not better than HE4 and CPH-I.

Moreover, studies showed that similar pathological subtypes of ovarian tumors were more difficult to distinguish as similar imaging [30], and the four indices were different among different pathological subtypes [20, 21]. Therefore, we compared the four indices and their performance for distinguishing the corresponding pathological subtypes of EOC I+II from BOT I+II. We found that the HE4 levels and CPH-I values in the serous EOC I+II subgroups were much higher than that of BOT I+II subgroups and the AUC of both HE4 and CPH-I were significantly bigger than that of CA125. However, in mucinous EOC I+II, though the HE4 levels and ROMA and CPH-I values were higher than that of BOT I+II subgroups, the AUC among them had no significant difference, and in the endometrioid subgroups, there were no significant differences in the levels or AUC of the four indices. These meant that the HE4 and CA125 levels and ROMA and CPH-I values were affected by pathological subtypes. The four indices were all not suitable for differentiating BOT I+II and EOC I+II of the endometrioid type, while HE4 and CPH-I were more favorable than CA125 in differentiating BOT I+II and EOC I+II of serous types. Therefore, both HE4 and CPH-I might have wider applicability than CA125 in the case of unknown pathology.

Our study has a relatively large sample size with 144 BOT and 108 EOC participants in FIGO stages I and II, and we excluded almost all the participants with any influencing factors. Therefore, our study is representative in differentiating BOT from EOC in early stages. However, our research has a monocentric design and is a retrospective study which might bias the results. What is more, the statistical analysis of the four indices of the clear cell tumors was not performed as there were only four clear cell BOT I+II cases. So, the findings of this study should be confirmed in wider populations.

In conclusion, CPH-I was more valuable than CA125 as a predictive biomarker for differentiating borderline ovarian tumors from epithelial ovarian cancer in early stages regardless of menopausal status, while HE4 might be better than CA125 for postmenopausal subgroups. Both HE4 and CPH-I were more favorable than CA125 for differentiating BOT I+II and EOC I+II in the case of unknown pathology or in serous types. However, HE4, CA125, ROMA, and CPH-I were all not capable for differentiating BOT I+II from EOC I+II of endometrioid subtype.

## Figures and Tables

**Table 1:** Patient characteristics.

	Benign (*n* = 238)	BOT I+II (*n* = 144)	EOC I+II (*n* = 108)
Age (year)	32.0 (27.0-40.0)	32.0 (27.0-41.0)	48.0 (41.0-58.75)^∗^^^^
Premenopausal	218	122	61
Postmenopausal	20	22	47
FIGO stages			
I	—	140	76
II	—	4	32
Histopathology			
Serous	—	91	43
Mucinous	—	40	10
Endometrioid	—	9	23
Clear cell	—	4	32

Note: data of age are presented as the median (interquartile range). Abbreviations: *n* = number; FIGO = International Federation of Gynecology and Obstetrics; BOT I+II = borderline ovarian tumors in FIGO stages I and II; EOC I+II = epithelial ovarian cancer in FIGO stages I and II.

**Table 2:** Comparison of HE4, CA125, ROMA, and CPH-I in different groups.

	Benign			BOT I+II			EOC I+II		
	All	Pre-M	Post-M	All	Pre-M	Post-M	All	Pre-M	Post-M
HE4 (pmol/L)	43.8 (39.2-49.1)	43.9 (38.9-49.1)	43.5 (41.5-55.9)	48.4 (41.8-56.5)^∗^	47.6 (41.2-56.0)^∗^	50.1 (45.4-61.4)	80.3 (55.3-171.2)^∗^^^^	64.2 (50.7-136.4)^∗^^^^	95.0 (67.4-221.0)^∗^^^^
CA125 (U/mL)	24.9 (15.2-55.0)	25.3 (16.2-61.0)	13.6 (10.5-33.7)	34.1 (19.5-69.0)^∗^	35.6 (21.5-67.3)^#^	22.2 (16.9-189.8)	95.1 (35.6-234.5)^∗^^^^	88.5 (35.7-182.1)^∗^^^^	104.5 (33.4-320.7)^∗^
ROMA (%)	6.03 (4.5-8.0)	5.7 (4.4-7.4)	11.5 (8.1-16.5)	7.5 (5.3-12.0)^∗^	6.7 (5.1-10.3)^∗^	15.4 (11.7-56.5)	30.7 (10.6-65.6)^∗^^^^	14.6 (8.7-49.5)^∗^^^^	59.7 (28.6-81.5)^∗^^^^
CPH-I (%)	1.0 (0.7-2.3)	1.0 (0.7-2.2)	1.8 (0.9-2.5)	1.6 (0.9-3.1)^∗^	1.4 (0.9-2.8)^#^	2.3 (1.4-16.1)	11.3 (3.3-42.6)^∗^^^^	7.2 (2.3-26.0)^∗^^^^	25.1 (7.4-59.9)^∗^^^^

Note: data are presented as the median (interquartile range). ^∗^*P* < 0.01 and ^#^*P* < 0.05 compared with benign corresponding subgroups; ^^^*P* < 0.01 compared with BOT I+II corresponding subgroups. Abbreviations: Pre-M = premenopausal; Post-M = postmenopausal; BOT I+II = borderline ovarian tumors in FIGO stages I and II; EOC I+II = epithelial ovarian cancer in FIGO stages I and II.

**Table 3:** SN, SP, PPV, and NPV of CA125, HE4, ROMA, and CPH-I in differentiating BOT I+II from EOC I+II group stratification by different menopausal statuses.

Menopausal status	SN (%)	SP (%)	PPV (%)	NPV (%)
All				
HE4	65.74	84.03	75.53	76.58
CA125	55.56	81.25	68.97	70.91
ROMA	62.96	88.19	46.45	75.61
CPH-I	78.7	74.31	69.70	82.30
Pre-M				
HE4	73.77	66.39	64.00	78.20
CA125	54.1	82.79	61.11	78.29
ROMA	78.69	64.75	63.23	85.71
CPH-I	70.49	78.69	62.30	84.20
Post-M				
HE4	70.21	90.91	88.64	68.00
CA125	76.6	63.64	81.82	44.44
ROMA	82.98	68.18	73.21	53.85
CPH-I	85.11	68.18	85.10	68.20

Abbreviations: SN = sensitivity; SP = specificity; PPV = positive predictive value; NPV = negative predictive value; Pre-M = premenopausal; Post-M = postmenopausal; BOT I+II = borderline ovarian tumors in FIGO stages I and II; EOC I+II = epithelial ovarian cancer in FIGO stages I and II.

**Table 4:** Comparison of HE4, CA125, ROMA, and CPH-I among BOT I+II and EOC I+II group stratification by pathology.

	BOT I+II				EOC I+II			
	Serous	Mucinous	Endometrioid	Clear cell	Serous	Mucinous	Endometrioid	Clear cell
HE4 (pmol/L)	49.8 (42.5-57.9)	45.3 (38.9-53.0)	57.4 (47.4-89.9)	37.8 (31.5-42.7)	130.6 (72.6-230.5)^∗^	57.3 (47.3-65.1)^∗^	91.9 (55.6-221.0)	64.8 (50.6-82.3)
CA125 (U/mL)	39.4 (19.6-86.5)	25.5 (17.9-37.2)	54.2 (24.8-127.3)	29.6 (22.7-53.5)	105.8 (55.5-392.9)^∗^	54.7 (16.0-117.8)	103.5 (32.1-251.8)	95.1 (34.5-180.3)
ROMA (%)	8.2 (5.5-12.5)	6.1 (4.2-9.6)	11.0 (7.0-43.8)	8.59 (3.0-18.4)	59.7 (23.5-81.9)^∗^	11.2 (7.1-14.0)^#^	45.1 (10.6-82.0)	21.2 (9.4-32.0)
CPH-I (%)	1.8 (1.2-4.6)	1.0 (0.6-2.0)	1.9 (1.4-11.4)	1.5 (0.9-2.3)	30.4 (7.8-56.1)^∗^	3.0 (1.7-7.9)^∗^	11.4 (2.5-57.0)	7.3 (3.1-14.6)

Note: data are presented as the median (interquartile range). ^∗^*P* < 0.01 and ^#^*P* < 0.05 EOC I+II vs. BOT I+II in corresponding subgroups. Abbreviations: BOT I+II = borderline ovarian tumors in FIGO stages I and II; EOC I+II = epithelial ovarian cancer in FIGO stages I and II.

**Table 5:** SN, SP, PPV, and NPV of CA125, HE4, ROMA, and CPH-I in differentiating BOT I+II from EOC I+II group stratification by pathology.

Menopausal status	SN (%)	SP (%)	PPV (%)	NPV (%)
Serous				
HE4	76.74	90.11	78.57	89.13
CA125	60.47	75.82	54.17	80.23
ROMA	79.07	86.81	73.91	89.77
CPH-I	83.72	74.73	61.00	90.70
Mucinous				
HE4	70.00	85.00	53.85	91.89
CA125	70.00	72.50	38.89	90.63
ROMA	70.00	80.00	53.33	91.43
CPH-I	90.00	67.50	40.90	96.40
Endometrioid				
HE4	39.13	100	100	39.13
CA125	52.17	77.78	85.71	38.89
ROMA	43.48	100	100	40.91
CPH-I	65.22	77.78	88.20	46.70

Abbreviations: SN = sensitivity; SP = specificity; PPV = positive predictive value; NPV = negative predictive value; BOT I+II = borderline ovarian tumors in FIGO stages I and II; EOC I+II = epithelial ovarian cancer in FIGO stages I and II.

## Data Availability

The data used to support the findings of this study are available from the corresponding authors upon request.

## References

[1] Kung F. Y. L., Tsang A. K. H., Yu E. L. M. (2019). Intraoperative frozen section analysis of ovarian tumors: a 11-year review of accuracy with clinicopathological correlation in a Hong Kong Regional hospital. *International Journal of Gynecologic Cancer*.

[2] Denewar F. A., Takeuchi M., Urano M. (2017). Multiparametric MRI for differentiation of borderline ovarian tumors from stage I malignant epithelial ovarian tumors using multivariate logistic regression analysis. *European Journal of Radiology*.

[3] Trimble E. L., Trimble C. L. (2001). Ovarian tumors of low malignant potential. *Current Treatment Options in Oncology*.

[4] Hauptmann S., Friedrich K., Redline R., Avril S. (2017). Ovarian borderline tumors in the 2014 WHO classification: evolving concepts and diagnostic criteria. *Virchows Archiv*.

[5] Mangili G., Somigliana E., Giorgione V. (2016). Fertility preservation in women with borderline ovarian tumours. *Cancer Treatment Reviews*.

[6] du Bois A., Trillsch F., Mahner S., Heitz F., Harter P. (2016). Management of borderline ovarian tumors. *Annals of Oncology*.

[7] Yazbek J., Ameye L., Timmerman D. (2010). Use of ultrasound pattern recognition by expert operators to identify borderline ovarian tumors: a study of diagnostic performance and interobserver agreement. *Ultrasound in Obstetrics & Gynecology*.

[8] Yazbek J., Raju K. S., Ben-Nagi J., Holland T., Hillaby K., Jurkovic D. (2007). Accuracy of ultrasound subjective ‘pattern recognition’ for the diagnosis of borderline ovarian tumors. *Ultrasound in Obstetrics & Gynecology*.

[9] Bazot M., Nassar-Slaba J., Thomassin-Naggara I., Cortez A., Uzan S., Darai E. (2006). MR imaging compared with intraoperative frozen-section examination for the diagnosis of adnexal tumors; correlation with final histology. *European Radiology*.

[10] Di Lorenzo G., Ricci G., Severini G. M., Romano F., Biffi S. (2018). Imaging and therapy of ovarian cancer: clinical application of nanoparticles and future perspectives. *Theranostics*.

[11] Gizzo S., Berretta R., di Gangi S. (2014). Borderline ovarian tumors and diagnostic dilemma of intraoperative diagnosis: could preoperative He4 assay and ROMA score assessment increase the frozen section accuracy? A multicenter case-control study. *BioMed Research International*.

[12] Kim J. H., Kim T. J., Park Y. G. (2009). Clinical analysis of intra-operative frozen section proven borderline tumors of the ovary. *Journal of Gynecologic Oncology*.

[13] Chen F., Shen J., Wang J., Cai P., Huang Y. (2018). Clinical analysis of four serum tumor markers in 458 patients with ovarian tumors: diagnostic value of the combined use of HE4, CA125, CA19-9, and CEA in ovarian tumors. *Cancer Management and Research*.

[14] Abdalla N., Piorkowski R., Bachanek M., Stanirowski P., Cendrowski K., Sawicki W. (2018). Does the risk of ovarian malignancy algorithm provide better diagnostic performance than HE4 and CA125 in the presurgical differentiation of adnexal tumors in Polish women?. *Disease Markers*.

[15] Minar L., Felsinger M., Cermakova Z., Zlamal F., Bienertova-Vasku J. (2018). Comparison of the Copenhagen Index versus ROMA for the preoperative assessment of women with ovarian tumors. *International Journal of Gynaecology & Obstetrics*.

[16] Lycke M., Kristjansdottir B., Sundfeldt K. (2018). A multicenter clinical trial validating the performance of HE4, CA125, risk of ovarian malignancy algorithm and risk of malignancy index. *Gynecologic Oncology*.

[17] Karlsen M. A., Høgdall E. V. S., Christensen I. J. (2015). A novel diagnostic index combining HE4, CA125 and age may improve triage of women with suspected ovarian cancer - an international multicenter study in women with an ovarian mass. *Gynecologic Oncology*.

[18] Chen J., Chang C., Huang H. C. (2015). Differentiating between borderline and invasive malignancies in ovarian tumors using a multivariate logistic regression model. *Taiwanese Journal of Obstetrics & Gynecology*.

[19] Yoshida A., Derchain S. F., Pitta D. R., de Angelo Andrade L. A. L., Sarian L. O. (2016). Comparing the Copenhagen Index (CPH-I) and Risk of Ovarian Malignancy Algorithm (ROMA): two equivalent ways to differentiate malignant from benign ovarian tumors before surgery?. *Gynecologic Oncology*.

[20] Zhang L., Chen Y., Wang K. (2019). Comparison of CA125, HE4, and ROMA index for ovarian cancer diagnosis. *Current Problems in Cancer*.

[21] Zheng L. E., Qu J. Y., He F. (2016). The diagnosis and pathological value of combined detection of HE4 and CA125 for patients with ovarian cancer. *Open Medicine*.

[22] Moore R. G., McMeekin D. S., Brown A. K. (2009). A novel multiple marker bioassay utilizing HE4 and CA125 for the prediction of ovarian cancer in patients with a pelvic mass. *Gynecologic Oncology*.

[23] Moore R. G., Jabre-Raughley M., Brown A. K. (2010). Comparison of a novel multiple marker assay vs the Risk of Malignancy Index for the prediction of epithelial ovarian cancer in patients with a pelvic mass. *American Journal of Obstetrics and Gynecology*.

[24] Chudecka-Glaz A., Cymbaluk-Ploska A., Luterek-Puszynska K., Menkiszak J. (2016). Diagnostic usefulness of the Risk of Ovarian Malignancy Algorithm using the electrochemiluminescence immunoassay for HE4 and the chemiluminescence microparticle immunoassay for CA125. *Oncology Letters*.

[25] Karlsen M. A., Sandhu N., Høgdall C. (2012). Evaluation of HE4, CA125, risk of ovarian malignancy algorithm (ROMA) and risk of malignancy index (RMI) as diagnostic tools of epithelial ovarian cancer in patients with a pelvic mass. *Gynecologic Oncology*.

[26] Kotowicz B., Fuksiewicz M., Sobiczewski P. (2015). Clinical value of human epididymis protein 4 and the Risk of Ovarian Malignancy Algorithm in differentiating borderline pelvic tumors from epithelial ovarian cancer in early stages. *European Journal of Obstetrics, Gynecology, and Reproductive Biology*.

[27] Coburn S. B., Bray F., Sherman M. E., Trabert B. (2017). International patterns and trends in ovarian cancer incidence, overall and by histologic subtype. *International Journal of Cancer*.

[28] Chen V. W., Ruiz B., Killeen J. L. (2003). Pathology and classification of ovarian tumors. *Cancer*.

[29] Fuh K. C., Java J. J., Chan J. K. (2019). Differences in presentation and survival of Asians compared to Caucasians with ovarian cancer: an NRG Oncology/GOG Ancillary study of 7914 patients. *Gynecologic Oncology*.

[30] Ramalingam P. (2016). Morphologic, immunophenotypic, and molecular features of epithelial ovarian cancer. *Oncology*.

